# Targeting INHBA in Ovarian Cancer Cells Suppresses Cancer Xenograft Growth by Attenuating Stromal Fibroblast Activation

**DOI:** 10.1155/2019/7275289

**Published:** 2019-11-11

**Authors:** Xiaoting Li, Zongyuan Yang, Sen Xu, Zhengzheng Wang, Ping Jin, Xin Yang, Zeyu Zhang, Ya Wang, Xiao Wei, Tian Fang, Qinglei Gao

**Affiliations:** ^1^Cancer Biology Research Center (Key Laboratory of the Ministry of Education), Tongji Hospital, Tongji Medical College, Huazhong University of Science and Technology, Wuhan, Hubei 430030, China; ^2^Department of Hepatobiliary and Pancreatic Surgery, The Affiliated Tumor Hospital of Zhengzhou University, Henan Cancer Hospital, Zhengzhou, Henan 450008, China; ^3^Department of Obstetrics and Gynecology, The Central Hospital of Wuhan, Tongji Medical College, Huazhong University of Science and Technology, Wuhan, Hubei 430030, China

## Abstract

INHBA-encoded inhibin *β* A is a member of the transforming growth factor-*β* (TGF-*β*) superfamily. INHBA has been reported to be implicated in the progression of multiple types of cancer including ovarian cancer (OC). However, the mechanisms by which INHBA affects OC progression are not well-characterized. The aim of our study was to explore the prognostic value of INHBA for different stages and grades of OC and to identify the possible mechanisms by which INHBA promotes OC progression. Our results demonstrated that INHBA was specifically expressed in OC epithelium, and higher expression was associated with higher risk of mortality in patients with advanced and higher-grade serous OC (SOC). In addition, knockdown of INHBA in cancer cells impaired cancer xenograft growth through reducing OC stromal fibroblast activation *in vivo.* Further results confirmed that Smad2 signaling pathway was involved in INHBA-induced stromal fibroblast activation, and inhibiting this pathway could effectively reverse activation of stromal fibroblasts. In summary, our results showed that blocking INHBA in cancer cells may be a potential therapeutic strategy to inhibit SOC progression.

## 1. Introduction

Ovarian cancer (OC) is the seventh leading cancer diagnosis and eighth leading cause of cancer death among women [1]. OC is highly curable if found early and intervened actively, but OC at early stage usually lacks obvious clinical symptoms. Around 60-70% of women are diagnosed with late-stage disease that has already spread within the abdomen [2, 3]. Despite numerous targeted drugs have been developed to treat OC, patients' overall survival (OS) is still very dismal [4]. Therefore, it is urgent and significant to identify novel molecules involved in the OC progression and further develop some other effective treatments for OC patients.

INHBA-encoded inhibin *β* A is a member of the transforming growth factor-*β* (TGF-*β*) superfamily [5]. Inhibin *β* A could further form activin A by homodimerization or be linked to inhibin *β* B to produce inhibin by heterodimerization [6]. Activin A has been reported to be involved in a variety of biological processes, such as immune response, stem cell differentiation, and glucose metabolism [7]. Recent studies have shown that overexpression of INHBA occurs in multiple types of cancers, including colorectal cancer, breast cancer, lung cancer, esophageal squamous cell carcinoma, and bladder cancer [6, 8–11]. For example, activin A signaling promotes breast cancer metastasis by regulating IL13R*α*2 expression [8]. In esophageal squamous cell carcinoma, high INHBA level predicts poorer prognosis [10]. Upregulation of INHBA expression promotes cell proliferation and predicts poor survival in patients with lung adenocarcinoma [6]. In OC, although some studies have reported that patients with high expression of INHBA have shorter survival times than those with low expression of INHBA [12], the relationship of INHBA with clinical features and its contribution to OC progression have not been fully elucidated.

Overwhelming studies have demonstrated the important contribution of stromal involvement to the pathogenesis of OC [13, 14]. As the predominant cell type in the cancer stromal compartment, cancer-associated fibroblasts (CAFs) are reported to actively promote migration and invasion of tumor cells and impede drug delivery through generating extracellular matrix (ECM) components [15–18]. Currently, activated CAFs were considered to mainly originate from the surrounding fibroblasts under education of cancer cells [19, 20]. The ability of tumor cells to direct normal fibroblasts to differentiate into CAFs is dependent on cytokines such as PDGF, TGF *β*1, and FGF2 [21]. Recent studies have reported that INHBA is also a driver of the CAF phenotype in OC [22, 23]. However, our knowledge regarding activation effect of INHBA on fibroblasts is remained insufficient.

Therefore, the purposes of this study were to evaluate the expression of INHBA in OC tissues and to characterize the pivotal role of cancer cell-derived INHBA in stromal fibroblast activation and SOC progression. This study could help us better understand INHBA-mediated interaction between cancer cells and stromal fibroblasts, providing evidence to support that targeting INHBA in cancer cells to inactivate stromal fibroblasts could be a promising SOC therapeutic strategy.

## 2. Methods

### 2.1. Cell Culture

We purchased human ovarian cancer cell lines (SKOV3, CAROV3, OVCAR8, and OV90) and fibroblast cell line MRC-5 from ATCC (Rockville, MD, USA) and the cell bank of the Chinese Academy of Sciences, respectively. All the cell lines were confirmed to be mycoplasma-free by the source organizations prior to purchase. We isolated and purified primary normal ovarian fibroblasts (NOFs) from OC patients normal fresh ovary tissues following procedures as previously described [24]. Briefly, all the patient tissues were obtained under the supervision of the Ethics Committee of Tongji Hospital and confirmed by two senior pathologists. To collect tissue homogenate, fresh normal ovary tissues of 1 mm^3^ were digested on a shaker in serum-free DMEM/F-12 containing collagenase and hyaluronidase (Sigma) for 2-3 hours. After the termination of digestion with FBS (Gibco), all the tissue samples were incubated with red blood cell lysate (BioLegend), filtered using a 40 *μ*m cell strainer (BD Biosciences) to obtain single cells, and then subjected to antifibroblast microbeads (Miltenyi Biotec; 130-050-601) for fibroblast isolation. Finally, single cell samples were sorted using an MACS column (Miltenyi Biotec). The quality of the NOFs was confirmed using a PDGFR*α* antibody (ab203491, Abcam, USA). All OC cell lines were cultured in McCoy's 5A medium, and MRC-5 and the primary NOFs were maintained in DMEM/F-12 medium. All the cells were cultured in an incubator at 37°C, 5% CO_2_, and 80% humidity. All of aforementioned media were mixed with 1% penicillin/streptomycin (Thermo Scientific) and 10% FBS (Gibco).

### 2.2. Public Database Analysis

We used Oncomine online tool (https://www.oncomine.org) to examine INHBA expression in microdissected ovarian profile GSE26712 and TCGA dataset. Student's *t*-test was used to calculate statistical significance. Gene expression data (GSE26193, GSE9891, GSE51088 profiling data) were obtained from Gene Expression Omnibus online website (https://www.ncbi.nlm.nih.gov/geo). The TCGA expression dataset and coexpression genes with INHBA were obtained via the cBioPortal tools (http://cbioportal.org). Screening criteria was based upon Spearman's correlation. The David analysis tool (https://david.ncifcrf.gov) and the Kobas analysis tool (http://kobas.cbi.pku.edu.cn) were used to perform the GO analysis and the KEGG pathway analysis.

### 2.3. Single-Sample Gene Set Enrichment Analysis (ssGSEA)

In order to investigate the relationship between INHBA expression and the 141-stroma signature activation degree in GSE9891, GSE51088, GSE26193, and TCGA dataset, ssGSEA was used to generate geneset activation score as described previously [25].

### 2.4. Tissue Sample Information

Tissue samples in this study consisted of 224 cases from two sources. The first source was a tissue microarray obtained from US Biomax authorized AlenaBio (OV2084a; https://www.alenabio.com). There were 208 samples included in the tissue microarray, which included formalin-fixed paraffin-embedded serous papillary adenocarcinoma (*n* = 130), serous adenocarcinoma (*n* = 2), adenocarcinoma (*n* = 7), endodermal sinus carcinoma (*n* = 7), mucinous papillary adenocarcinoma (*n* = 24), dysgerminoma (*n* = 5), endometrioid carcinoma (*n* = 3), immature teratoma (*n* = 2), embryonal carcinoma (*n* = 1), mature teratoma (*n* = 1), clear cell carcinoma (*n* = 1), transitional cell carcinoma (*n* = 1), strumal carcinoid (*n* = 1), squamous cell carcinoma from teratoma with malignant transformation (*n* = 3), granular cell tumor (*n* = 4), normal ovarian epithelial tissue (*n* = 2), and adjacent normal ovary tissue (*n* = 14). Clinical data such as age, histological type, differentiation, FIGO stage, and other information were also obtained from AlenaBio. Another source of tissue samples was the Department of Pathology of Tongji Hospital. We applied for and obtained 16 normal ovarian tissue sections from different patients under the supervision of the Ethics Committee of Tongji Hospital.

### 2.5. Immunohistochemistry, Masson's Trichrome Staining, and Picrosirius Red Staining

Immunohistochemistry was performed on paraffin-embedded tissue sections. The sections were first deparaffinized and then gradually hydrated. Antigen retrieval was performed by pressure cooking in 0.01 M citrate buffer for 10 min. Then, sections were incubated with 20% normal goat serum for 30 min at 37°C. Next, the slides were incubated with primary antibodies against INHBA (Proteintech, USA), FAP(ab28244, Abcam, USA), *α*-SMA(ab5694, Abcam, USA), FSP1(ab197896, Abcam, USA), and Ki-67(ab16667, Abcam, USA) at a dilution of 1 : 100 and then incubated with horseradish peroxide-conjugated secondary antibody for 20 min. Finally, a DAB kit (BD Biosciences) was used for the slides to obtain the best staining intensity. Immunostaining score was evaluated by 2 independent investigators who were unaware of the sections' information using a semiquantitative scoring system. Briefly, the intensity of immunostaining for INHBA is graded as follows: 0 = negative, 1 = weak, 2 = moderate, and 3 = strong, and proportion of immunopositive cells is graded as follows: 0 = ≤4%, 1 = >5 to ≤ 25%, 2 = >25 to ≤ 50%, 3 = >50 to ≤ 75%, and 4 = >75%to ≤ 100%. Then, the 2 scores of the corresponding sample were integrated to obtain the final staining index. Points = 0 were marked as -, 1-4 points as +, 5-8 points as ++, and 9-12 points as +++. For statistical analysis, ++ and +++ were considered as high expression of INHBA, whereas - and + were considered as low expression. Masson's trichrome (Servicebio, Wuhan, China) staining and picrosirius red staining (Servicebio, Wuhan, China) were performed on paraffin-embedded sections of xenografts according to the corresponding manufacturer's protocol.

### 2.6. Quantitative Real-Time- (RT-) PCR

Total RNA of cells was isolated with TRIzol Reagents (Invitrogen) according to the standard protocol [26]. Reverse transcription of 2 *μ*g RNA was performed using random primers and M-MLV reverse transcriptase (Takara, Japan). RT-PCR reactions were carried out using the Bio-Rad CFX96 system with IQ SYBR Green supermix (Bio-Rad, Hercules, CA). Relative expression levels of interesting genes were analyzed using the *ΔΔ* Cq method [27]. GAPDH served as the internal control. The primer sequences of INHBA are as follows: forward, 5′-ACACAACAACTTTTGCTGCC-3′, and reverse, 5′-TCGTGTCACCACTGTCTTCTC-3′. The primer sequences of GAPDH are as follows: forward, 5′-ACCCATCACCATCTTCCAGGAG-3′, and reverse, 5′-GAAGGGGCGGAGATGATGAC-3′.

### 2.7. Transfection of siRNA and Lentivirus

For transient endogenous INHBA knockdown, the cells were transfected with INHBA-specific siRNA (si-INHBA) (RiboBio, Guangzhou, China) using Lipofectamine 3000 reagent (Invitrogen). Negative control (si-Ctrl) was used as a transfection control. In contrast, lentivirus targeting INHBA (sh-INHBA) (Vigene Biosciences, Shandong, China) was used for long-term INHBA knockdown, the transfection step was performed according to the manufacturer's instructions. Negative control (sh-Ctrl) was used as a transfection control. Specific human INHBA shRNA sequence was as follows: CCAAC-AGGACCAGGACCAA.

### 2.8. Western Blotting

Cellular proteins were dissolved in modified RIPA buffer. Modified RIPA buffer formula was as follows: 0.25% sodium deoxycholate, 50 mmol/l Tris-Cl (pH 7.4), 1% NP-40, 1 mmol/l ethylenediaminetetraacetic acid (EDTA), 1 mmol/l sodium fluoride (NaF), 150 mmol/l sodium chloride (NaCl), and 1 mmol/l phenylmethylsulfonyl fluoride (PMSF). The concentration of proteins was quantified by a bicinchoninic acid (BCA) assay (Thermo Scientific). A total of 40 *μ*g of protein was separated by SDS-PAGE and subsequently transferred to nitrocellulose membranes. After blocking in TBST buffer, the membrane was incubated with appropriate dilutions of primary antibodies overnight at 4°C: INHBA (Proteintech, USA), GAPDH (Proteintech, USA), FAP (ab28244, Abcam, USA), *α*-SMA (ab5694, Abcam, USA), FSP1 (ab197896, Abcam, USA), total Smad2 (ab33875, Abcam, USA), and p-Smad2(Ser 465/467) (#18338, Cell Signaling Technology). Next day, the membrane was incubated with the corresponding peroxidase-conjugated secondary antibodies (Antgene). Finally, the enhanced chemiluminescence (ECL, Bio-Rad) was used to detect the antigen-antibody complexes. GAPDH was used as the control [23].

### 2.9. Immunofluorescence Staining

The cells were grown on cover slips. Next day, the cells were fixed with paraformaldehyde for 20 min, then rinsed three times with phosphate buffered saline (PBS), and followed by incubation with PBS containing 5% bovine serum albumin (BSA) and 0.1% Triton X-100 to permeabilize and block nonspecific antibody binding. Then, the samples were incubated with primary antibodies, including *α*-SMA (ab5694, Abcam, USA) and Ki-67 (ab16667, Abcam, USA) overnight at 4°C. Third day, the cells were incubated with secondary antibody (Alexa Fluor 594 conjugated, Antgene), phalloidin-iFluor 488 Reagent (ab176753, Abcam, USA), and DAPI (Sigma). Fluorescence images were obtained using an Olympus BX53 microscope (Olympus, Tokyo, Japan).

### 2.10. Collagen Gel Contraction Assay

Briefly, 6 × 10^5^ cells were prepared in each group and washed with PBS. Then, the cells were resuspended in 200 *μ*l collagen gel mixture per well in a 96-well ultra-low adhesion plate (Corning Life Sciences, Corning, NY). 200 *μ*l of collagen gel mixture formula was as follows: 31.25 *μ*l Type 1 Rat Tail Collagen (Thermo), 168.75 *μ*l DMEM/F-12 medium, and 0.72 *μ*l 1 N NaOH. After 12 h, the images were taken and contraction area was quantified with ImageJ software. All contraction assays were performed in triplicate.

### 2.11. Cellular Viability Assay

Cells were seeded in 24-well plates in triplicate with an initial density of 1 × 10^4^ cells per well. At 24 h, 48 h, 72 h, and 96 h since planting, the cells were digested with trypsin and counted using an automated cell counter.

### 2.12. Xenograft Tumor Model

All procedures performed in studies involving mice were carried out according to the Regulations for the Administration of Affairs Concerning Experimental Animals of China (2017) and were approved by the Committee on the Ethics of Animal Experiments in the Hubei Province. Ten female BALB/c nude mice (weight 20–23 g, 4-6 weeks of age) were purchased and cultured in laminar flow cabinets under specific pathogen-free conditions. The mice were randomly divided into two groups (*n* = 5 per group). 2 × 10^6^ SKOV3 tumor cells expressing sh-INHBA and sh-Ctrl were subcutaneously implanted in the right backs of the mice in the INHBA knockdown group and in the control group, respectively. All mice were killed humanely on day 28 after transplantation, and tumor nodules were dissected, weighed, and paraffin-embedded for subsequent detection.

### 2.13. Statistics

All statistical analyses were performed using SPSS 22.0 (IBM, Ehningen, Germany) and GraphPad Prism 5.0 software (GraphPad Inc., San Diego, CA, USA). The Shapiro-Wilk test was used to evaluate whether the data were normally distributed. For normally distributed data, the data were presented as means ± standard error of the mean (s.e.m.) for at least three independent experiments and evaluated using Student's unpaired two-tailed *t*-test for comparisons between two groups, and one-way ANOVA followed by Tukey's posttest for analyses among multiple groups. For nonnormally distributed data (INHBA IHC scores), the data were presented as medians ± interquartile range, and the Mann-Whitney test was performed to compare the IHC scores between the normal ovary and OC groups. The Kruskal-Wallis test and chi-squared test were used to analyze the relationship between INHBA expression and clinical pathological parameters. *P* < 0.001 was considered very significant (^∗∗∗^), *P* < 0.01 was considered highly significant (^∗∗^), and *P* < 0.05 was considered statistically significant (^∗^).

## 3. Results

### 3.1. INHBA Is Specifically Overexpressed in OC Epithelium

**Figure 1:**
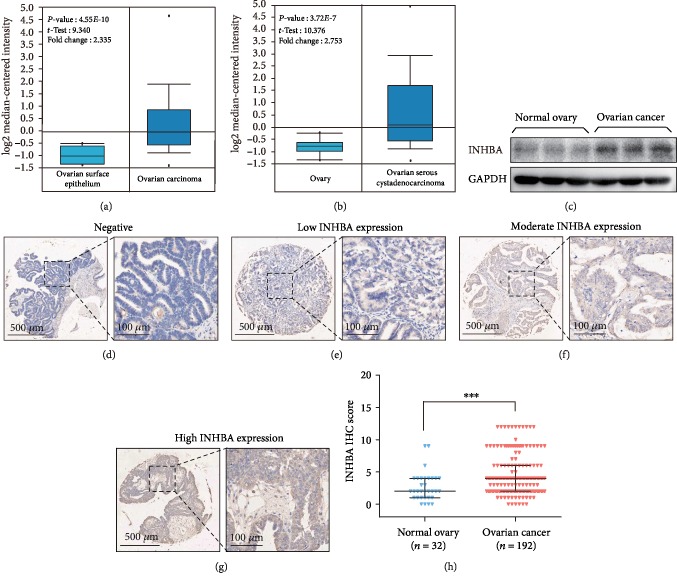
INHBA is specifically overexpressed in OC tissues. (a, b) Oncomine analysis showed the mRNA expression of INHBA was significantly increased in OC. (c) Western blot analysis of INHBA in normal ovary tissues and OC tissues. GAPDH was used as the loading control. (d–g) IHC staining of INHBA protein in OC: (d) negative INHBA staining, (e) low INHBA expression, (f) moderate INHBA expression, and (g) high INHBA expression. (h) Comparison of IHC scores from the normal ovary and OC groups. Data are expressed as mean ± s.e.m. or median ± interquartile range, ^∗^*P* < 0.05; ^∗∗^*P* < 0.01; ^∗∗∗^*P* < 0.001.

In order to investigate the expression pattern of INHBA in OC and normal ovary, we analyzed two microdissected profiles including OC and normal ovary samples and found that INHBA mRNA was elevated in the OC samples (Figures 1(a) and 1(b)). Immunoblotting analysis showed that INHBA protein expression was significantly increased in OC tissues compared to that in normal ovarian tissues (Figure 1(c)). Subsequently, immunohistochemistry (IHC) analysis was performed on 192 OC tissues and 32 normal ovarian tissues. Consistent with Figures 1(a)–1(c), INHBA expression was obviously elevated in OC tissues. In detail, positive INHBA staining was mainly distributed in the tumor tissue epithelium (Figures 1(d)–1(g)). Among the 192 OC tissues, 55 (28.6%) cases showed high expression of INHBA, and 137 (71.4%) cases demonstrated low expression of INHBA. In contrast, only 3 (9.4%) of 32 normal ovarian tissues showed high expression of INHBA (Figure 1(h)).

### 3.2. Correlation between INHBA Expression and Clinicopathological Features

Among the many types of OC, SOC is the most common and deadliest type, which accounts for over 80% of all epithelial OC cases [1], so we mainly investigated the role of INHBA in SOC. To better understand the effect of INHBA on SOC progression, the relationship between INHBA expression and clinicopathological features was analyzed using the chi-squared test and Kruskal-Wallis test for 132 cases of SOC. As shown in Table 1, high INHBA expression in SOC was significantly associated with FIGO stage, and later stages of SOC had higher INHBA expression (*P* < 0.0001). Similarly, there was a tendency for positive correlation between INHBA expression and higher pathological grades (*P* = 0.039). However, we did not find significant correlation between INHBA expression and age (*P* = 0.363). These results indicated that there was a positive correlation between INHBA expression and SOC progression.

### 3.3. Prognostic Value of Different INHBA Expressions in SOC

**Figure 2:**
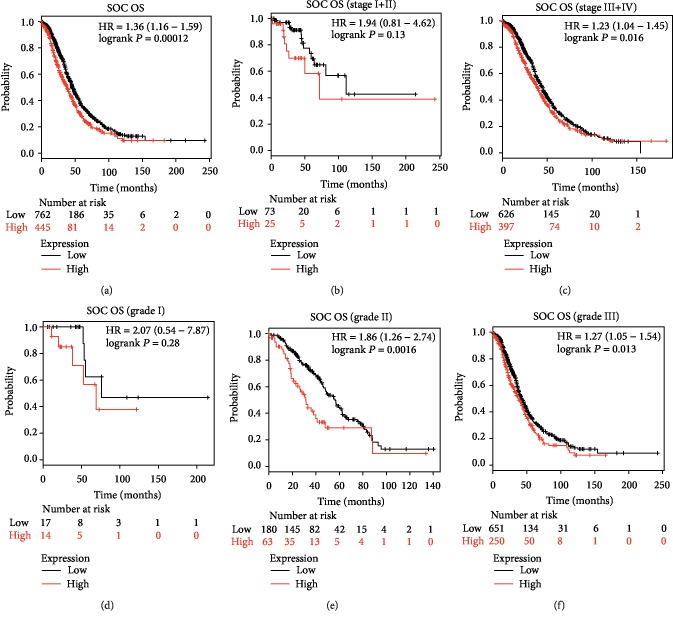
Prognostic value of different INHBA expressions in OS of SOC. Survival curves were plotted from KM plotter (https://kmplot.com). The desired Affymetrix ID was valid: 210511_s_at (INHBA). (a) For all SOC patients (*n* = 1207). (b) For all SOC patients of stage I+II (*n* = 98). (c) For all SOC patients of stage III+IV (*n* = 1023). (d) For all SOC patients of grade I (*n* = 31). (e) For all SOC patients of grade II (*n* = 243). (f) For all SOC patients of grade III (*n* = 901).

To investigate the relationship between INHBA expression and survival of patients with SOC, we then analyzed the prognostic value of INHBA for SOC by using an online tool (http://kmplot.com). Survival curves were plotted in http://kmplot.com for all patients with SOC (*n* = 1232), patients with stages I and II SOC (*n* = 98), patients with stages III and IV SOC (*n* = 1023), patients with grade I SOC (*n* = 31), patients with grade II SOC (*n* = 243), and patients with grade III SOC (*n* = 901). The Affymetrix ID was valid: 210511_s_at (INHBA). As depicted in Figure 2, high INHBA mRNA expression was correlated with worse overall survival (OS) of SOC patients [hazard ratio (HR) = 1.36 (1.16 − 1.59), *P* = 0.00012] (Figure 2(a)). More importantly, high expression of INHBA was associated with poor OS in patients with stages III and IV SOC [hazard ratio (HR) = 1.23 (1.04 − 1.45), *P* = 0.016], but not in patients with stages I and II SOC [hazard ratio (HR) = 1.94 (0.81 − 4.62), *P* = 0.13] (Figures 2(b) and 2(c)). Similarly, high expression of INHBA was correlated with poor survival prognosis in patients with grade II SOC [hazard ratio (HR) = 1.86 (1.26 − 2.74), *P* = 0.0016] and grade III SOC [hazard ratio (HR) = 1.27 (1.05 − 1.54), *P* = 0.013], but not in patients with grade I SOC [hazard ratio (HR) = 2.07 (0.54 − 7.87), *P* = 0.28] (Figures 2(d)–2(f)).

### 3.4. INHBA Blockade in Tumor Cells Does Not Significantly Inhibit Cellular Growth Ability *In Vitro*

**Figure 3:**
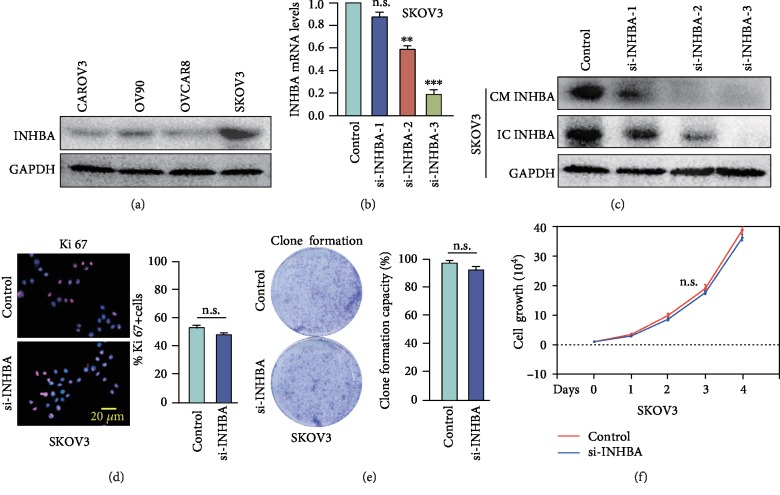
INHBA blockade in SKOV3 does not significantly inhibit cellular growth ability *in vitro*. (a) INHBA expression levels were measured by western blotting in 4 different human SOC cell lines. (b, c) The mRNA expression and protein expression of INHBA of SKOV3 cells in the absence or presence of si-INHBA. (d) Representative fluorescent images and statistical analysis of the relative number of Ki-67-labeled SKOV3 in the absence or presence of si-INHBA. (e) Representative images and statistical analysis of clonal formation ability of SKOV3 in the absence or presence of si-INHBA. (f) Cell counting assays detecting relative cell numbers of SKOV3 in the control or si-INHBA groups for various duration (1-4 days). Data are expressed as mean ± s.e.m., ^∗^*P* < 0.05; ^∗∗^*P* < 0.01; ^∗∗∗^*P* < 0.001.

Previous studies have shown that INHBA was mainly expressed in OC epithelium [28], and our IHC results confirmed this finding. To explore the biological mechanisms that could account for the association between high INHBA expression and poor survival, a series of experiments were carried out. Firstly, we evaluated INHBA expression levels in four SOC cell lines (Figure 3(a)) and then performed transient gene knockdown in SKOV3 that had a higher expression of INHBA. Real-time PCR and western blotting confirmed that si-INHBA not only efficiently suppressed the intracellular INHBA protein expression but also decreased extracellular INHBA protein levels in conditioned medium (CM) (Figures 3(b) and 3(c)). However, cell immunofluorescence for Ki-67 indicated that there were no significant differences in the positive staining rate between the si-INHBA group and control group (Figure 3(d)). Clone formation and cell counting assays showed that knockdown of INHBA did not significantly inhibit SKOV3 cellular growth ability (Figures 3(e) and 3(f)). Collectively, the above results confirmed that knockdown of INHBA has no noteworthy effect on the proliferation of SKOV3 *in vitro*.

### 3.5. INHBA Blockade in Tumor Cells Causes Xenograft Tumor Growth Inhibition and Reduces Stroma Components *In Vivo*

**Figure 4:**
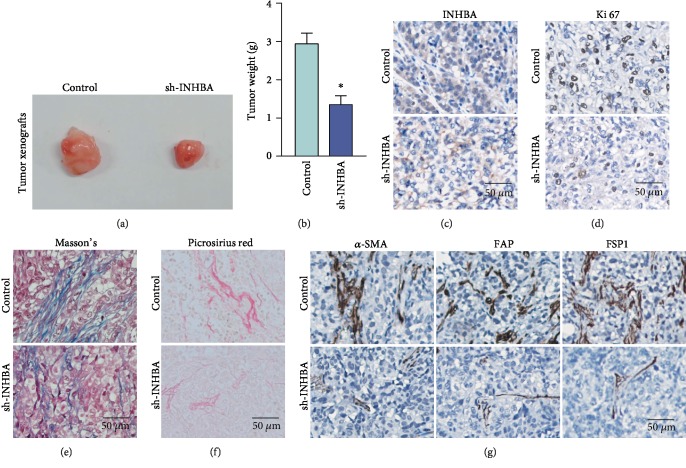
INHBA blockade in SKOV3 causes xenograft tumor growth inhibition and reduces stroma activation *in vivo*. (a, b) Bright field images and weight quantification of subcutaneous tumors from mice (*n* = 5) of the sh-Ctrl or sh-INHBA groups. (c) Representative IHC images of INHBA protein expression in the sh-INHBA-treated tumor tissues and sh-Ctrl tumor tissues. (d) Representative IHC images of Ki-67 protein expression in the sh-INHBA-treated tumor tissues and sh-Ctrl tumor tissues. (e, f) Masson's trichrome staining and picrosirius red staining in tumors from mice of the sh-Ctrl or sh-INHBA groups. (g) Representative IHC images of FAP, *α*-SMA, and FSP1 protein expression in tumors from mice of the sh-Ctrl or sh-INHBA groups. Data are expressed as mean ± s.e.m., ^∗^*P* < 0.05; ^∗∗^*P* < 0.01; ^∗∗∗^*P* < 0.001.

INHBA encodes Inhibin *β* A which is a member of the TGF-*β* pathway and has been declared to be a driver of the CAF phenotype in OC [22, 23]. Thus, considering the obvious prognostic significance of INHBA in SOC and limitations of *in vitro* experiments, we evaluated the role of INHBA in tumorigenesis *in vivo* using an immunodeficient mouse subcutaneous tumor model. Interestingly, treatment with sh-INHBA in SKOV3 cells obviously inhibited tumor growth *in vivo* and resulted in smaller tumors compared to the control group (Figures 4(a) and 4(b)). Further IHC analysis confirmed the *in vivo* stability of INHBA knockdown in SKOV3 (Figure 4(c)). More importantly, positive staining of Ki-67 was also obviously reduced in sh-INHBA-treated tumor xenografts (Figure 4(d)). Furthermore, Masson's trichome staining and picrosirius red staining showed that INHBA knockdown in SKOV3 cells resulted in downregulation of stromal components and reduced collagen deposition (Figures 4(e) and 4(f)). Besides, markers of fibroblast activation, such as *α*-SMA, FAP, and FSP1, were also obviously decreased in sh-INHBA-transduced tumor xenografts (Figure 4(g)). These above results demonstrated that blocking INHBA in SKOV3 may hamper tumorigenesis by reducing tumor stromal microenvironment activation *in vivo*.

### 3.6. INHBA from Tumor Cell-Derived Conditioned Medium (CM) Promotes Activation of Stromal Fibroblasts

**Figure 5:**
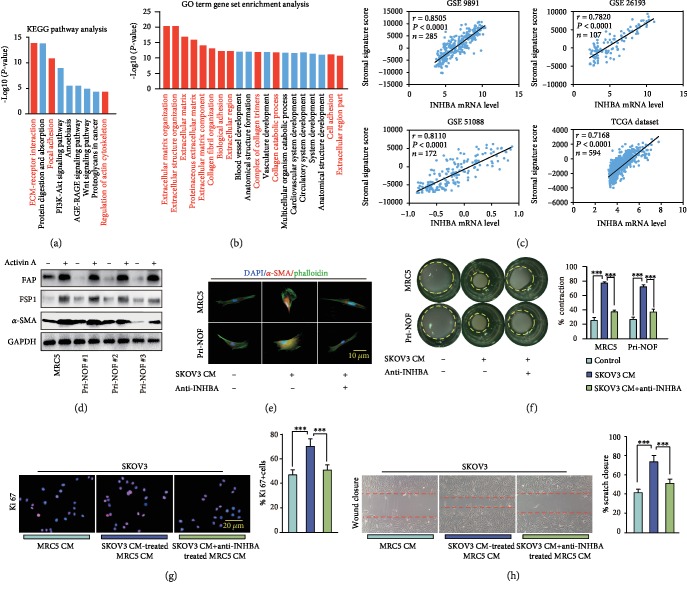
INHBA from SKOV3 CM promotes activation of stromal fibroblasts. (a, b) The GO term gene set enrichment analysis and KEGG pathway analysis using genes related with INHBA (Spearman′s correlation ratio > 0.8). (c) Spearman's correlation analysis showing the relation between the calculated stromal component score and expression level of INHBA in the GSE 9891, GSE 26193, GSE 51088, and TCGA dataset. (d) The results of western blot analysis showed that activin A enhanced the expression of fibroblast activation markers, such as FAP, *α*-SMA, and FSP1. GAPDH served as loading control. (e) In MRC5 and primary NOFs, an INHBA-neutralizing antibody suppressed the *α*-SMA elevation and cytoskeletal stretch caused by CM from SKOV3. (f) The INHBA-neutralizing antibody reversed the increased ability of fibroblasts to contract ECM caused by CM from SKOV3. (g, h) The immunofluorescence assay of Ki-67 and wound closure assay showing that INHBA-neutralizing antibody reversed the tumor-promoting ability of fibroblasts treated by CM from SKOV3. Data are expressed as mean ± s.e.m., ^∗^*P* < 0.05; ^∗∗^*P* < 0.01; ^∗∗∗^*P* < 0.001.

In order to better understand the promotion effect of INHBA on tumor growth *in vivo*, a series of experiments were subsequently conducted. Firstly, 23 genes coexpressed (Spearman′s correlation ratio > 0.8) with INHBA such as FAP, THBS2, COL5A2, VCAN, and COL11A1 were screened by using an online website (http://www.cbioportal.org) in the TCGA database. The KEGG and GO analyses showed that these genes related to INHBA were highly enriched in pathways that encoded ECM processes, such as extracellular matrix organization, extracellular structure organization, and collagen fibril organization (Figures 5(a) and 5(b)). Next, single-sample GSEA (ssGSEA) showed that INHBA mRNA expression was positively associated with the stromal activation score in the GSE 9891, GSE 26193, GSE 51088, and TCGA datasets (Figure 5(c)). Western blot data showed that exposure of fibroblasts to activin A obviously increased the expression of FAP, *α*-SMA, and FSP1 (Figure 5(d)). Furthermore, an INHBA-neutralizing antibody significantly reversed the *α*-SMA elevation and cytoskeletal stretch caused by SKOV3 CM in fibroblasts (Figure 5(e)). In addition, SKOV3 CM-induced upregulated ability of stromal fibroblasts in contracting ECM was also reduced by INHBA-neutralizing antibody (Figure 5(f)). Meanwhile, CM from SKOV3 CM-treated fibroblasts in turn increased the Ki-67-positive rate and cell growth in SKOV3, and these effects were eliminated by INHBA-neutralizing antibody (Figures 5(g) and 5(h)). These data implied that SKOV3 cell-derived INHBA promoted stromal fibroblast activation, and these activated fibroblasts maintained tumor cell growth.

### 3.7. The SMAD2 Signaling Pathway Is Involved in INHBA-Induced Fibroblast Activation

**Figure 6:**
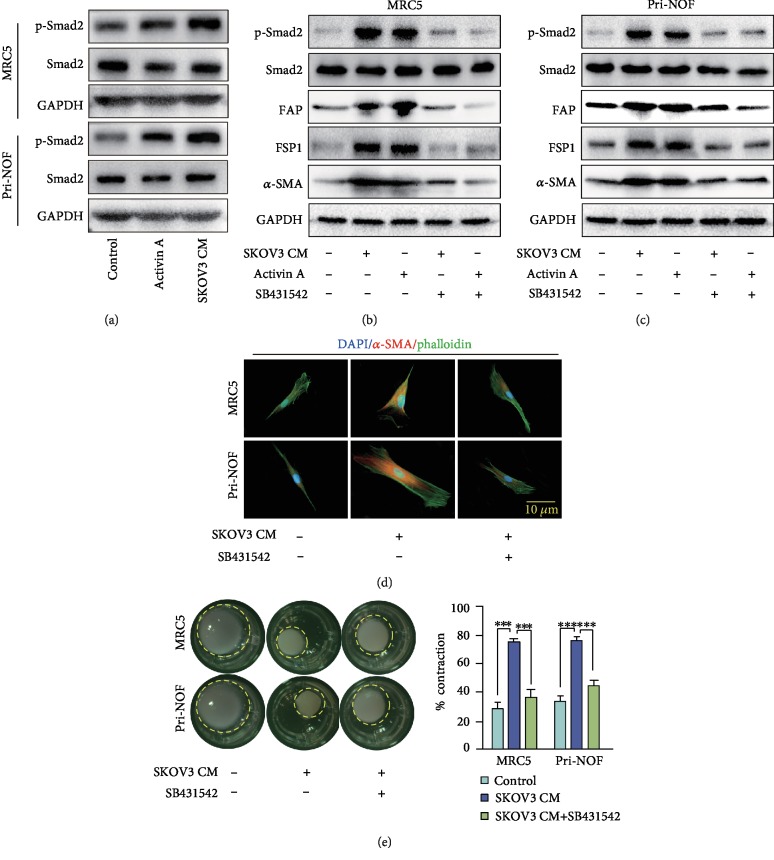
The Smad2 signaling pathway is involved in INHBA-induced fibroblast activation. (a) Immunoblotting showed that activin A or CM from SKOV3 increased the expression of p-Smad2 in fibroblast. GAPDH served as loading control. (b, c) SB431542 reduced the elevation of p-Smad2, FAP, *α*-SMA, and FSP1 expression in fibroblasts caused by activin A or CM from SKOV3. GAPDH served as loading control. (d) In MRC5 and primary NOFs, SB431542 suppressed the *α*-SMA increase and cytoskeletal stretch caused by CM from SKOV3. (e) SB431542 reversed the increased ability of fibroblasts to contract ECM caused by CM from SKOV3. Data are expressed as mean ± s.e.m., ^∗^*P* < 0.05; ^∗∗^*P* < 0.01; ^∗∗∗^*P* < 0.001.

It was well documented that INHBA is a member of the TGF-*β* pathway and can activate Smad signaling by binding to ACVR2A [5]. Similarly, our western blot analysis showed that SKOV3-derived CM and recombinant activin A both induced phosphorylation of Smad2 in NOFs and MRC5 (Figure 6(a)). Furthermore, SB431542, an inhibitor of the Smad signaling cascade, not only suppressed phosphorylation of Smad2 but also decreased stromal fibroblast activation induced by SKOV3-derived CM and recombinant activin A (Figures 6(b) and 6(c)). Immunofluorescence assay to evaluate *α*-SMA, and collagen contraction experiments, demonstrated that SB431542 effectively reversed stromal fibroblast activation caused by SKOV3-derived CM (Figures 6(d) and 6(e)). These data suggested that the Smad2 signaling pathway was involved in INHBA-induced fibroblast activation.

## 4. Discussion

**Figure 7:**
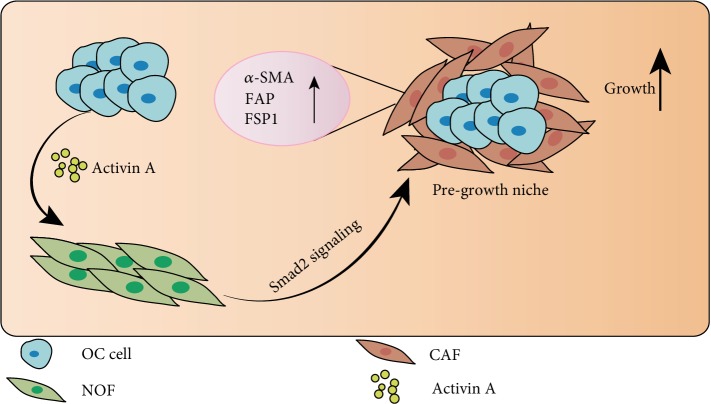
Schematic representation of the effect of INHBA derived from cancer cells on human ovarian stromal fibroblasts. INHBA from cancer cells triggered the Smad2 phosphorylation and induced activation of stromal fibroblasts, as evidenced by increased expression of fibroblast activation markers such as *α*-SMA, FAP, and FSP1. The activated stromal fibroblasts in turn promoted the growth of cancer cells and advanced cancer progression.

In the present study, we demonstrated that INHBA mRNA and protein were overexpressed in ovarian cancer (OC) tissues, and INHBA expression significantly increased with the advance of serous ovarian cancer (SOC) pathological grades and clinical stages. Kaplan-Meier plotter analysis showed that patients with SOC with higher INHBA expression had worse overall survival (OS) outcomes. Through comprehensive *in vivo* and *in vitro* experiments, we confirmed that knockdown of INHBA in tumor cells reduced OC stromal fibroblast activation, which turned to inhibit tumor growth. Furthermore, we found that the Smad2 signaling pathway was involved in INHBA-induced stromal fibroblast activation (Figure 7(a)).

Previous researches have demonstrated that the expression of INHBA is related with prognosis of different types of cancer, such as lung cancer, colorectal cancer, gastric cancer, urothelial carcinoma, and breast cancer [6, 8–11]. Okano et al. reported that INHBA was a predictor of poor prognosis in patients with colorectal cancer [9]. Wang et al. showed that INHBA overexpression implied adverse clinical outcomes in patients with gastric cancer [29]. In lung adenocarcinoma, Seder et al. demonstrated that upregulated expression of INHBA promoted cell proliferation and was related with poor survival [6]. In OC, INHBA was reported to cause sex cord-stromal tumors, which was evidenced by the occurrence of these tumors in INHA knockout mice [30]. However, the contribution of INHBA to epithelial OC progression is controversial. Dean et al. showed that OC patients with high INHBA expression had shorter survival times than patients with low expression of INHBA [12]. In contrast, Do et al. considered that INHBA expression in OC epithelia did not correlate with survival [31]. However, we speculated that this difference was due to the inconsistent OC stages and grades included in these studies. In our study, we analyzed the relationship between INHBA expression with pathological grades and clinical stages of patients with SOC, showing that INHBA expression significantly increased with the advance of SOC pathological grades and clinical stages. Upregulated expression of INHBA was linked with poorer OS in clinical stages III and IV as well as pathological grade II and III patients, but not in clinical stages I and II and pathological grade I patients.

The molecular mechanisms by which INHBA affects cancer progression remain elusive. Many studies have focused on metastasis and proliferation of tumor cells themselves. In non-small-cell lung cancer, INHBA has been shown to induce and maintain mesenchymal phenotypes of cancer stem-like cells and to promote cancer cell metastasis [32]. Additionally, INHBA gene is found to mediate activation of the TGF-*β* signaling pathway to promote gastric cancer cell migration and invasion [33]. In breast cancer, INHBA signaling promotes breast cancer metastasis by regulating IL13R*α*2 expression [8]. However, in OC, the role of INHBA is mixed and has not yet been fully elucidated. Welt et al. found that the majority of ovarian cancer cells did not exhibit accelerated proliferation in response to activin A treatment [34]. Ramachandran et al. showed that some epithelial OC cell lines did not respond to activin A, while others showed growth inhibition [35]. In our study, we demonstrated that INHBA was abundantly expressed in SKOV3 cells, and knockdown of INHBA did not significantly influence SKOV3 cellular growth ability *in vitro*.

However, OC cells grow in highly complicated stromal microenvironments that nurture them through metabolic remodeling, catabolism, autophagy, and inflammation and are capable of facilitating metastasis and resistance to therapy [36]. The results of *in vitro* experiments did not adequately represent the real situation *in vivo*. Our findings confirmed that decreased INHBA expression in SKOV3 had no effect on proliferation of tumor cells *in vitro*, but could hamper tumor xenografts growth by decreasing activation of stromal fibroblast *in vivo*. To better elucidate the regulation of stromal activation by INHBA in OC, we defined 23 genes coexpressed with INHBA including FAP, THBS2, COL5A2, VCAN, and COL11A1. Further analyses demonstrated that these 23 genes were mainly related to the stromal ECM and collagen-regulated processes. In addition, the stromal fibroblast activation caused by SKOV3-driven CM was significantly reversed by an INHBA-neutralizing antibody. Furthermore, we found that phosphorylation of Smad2 was involved in INHBA-induced fibroblast activation, and SB431542, an inhibitor of Smad signaling cascade, not only suppressed phosphorylation of Smad2 but also decreased stromal fibroblast activation induced by SKOV3-derived CM and recombinant activin A.

This study suffered from several limitations. First, single-source tumor tissue samples seem to be inadequate to reach greater reliability. Further multicentric and large-scale studies are required to verify our present findings. Second, the specific molecular mechanism by which INHBA activates stromal fibroblasts requires further characterization.

## 5. Conclusions

This study revealed the prognostic role of INHBA in SOC at different clinical stages and pathological grades. Additionally, our results shed light on the activation role of OC cell-derived INHBA in stromal fibroblasts, which was via the p-Smad2 pathway and promoted tumor xenograft growth. Our observations suggested that inhibition of INHBA in tumor cells could be a potential therapeutic approach to inhibit tumor progression and improve survival rates.

## Figures and Tables

**Table 1:** Association of INHBA expression with the clinicopathological variables of primary SOC patients (132^a^ cases).

Variable	INHBA expression			
	Low	High	Total	*P*
Age^a^				*P* = 0.363
≤50 years	29	18	47	
>50 years	56	27	83	
Stage^a^				*P* < 0.0001
I	81	31	112	
II	4	4	8	
III	0	6	6	
IV	0	4	4	
Grade^b^				*P* = 0.039
1	23	4	27	
2	9	4	13	
3	51	36	87	

^a^Two cases without epithelial components were excluded. ^b^Five cases without grade information were excluded.

## Data Availability

The data used to support this study are available from the corresponding author on reasonable request.

## Supplementary Materials

Supplementary Materials Supply Table 1: listing of genes associated with INHBA (Spearman′s correlation > 0.8) in ovarian serous cystadenocarcinoma (TCGA, provisional). Supply Table 2: KEGG pathway analysis with genes associated with INHBA. Supply Table 3: Gene Ontology analysis with genes associated with INHBA. Supply Table 4: listing of the genes included in the Yoshihara's 141-stromal signature (141 genes). Supply Table 5: ssGSEA scores of Yoshihara's 141-stromal signature in GSE9891, GSE26193, GSE51088, and TCGA dataset.
